# An Electromagnetic Sensor with a Metamaterial Lens for Nondestructive Evaluation of Composite Materials

**DOI:** 10.3390/s150715903

**Published:** 2015-07-03

**Authors:** Adriana Savin, Rozina Steigmann, Alina Bruma, Roman Šturm

**Affiliations:** 1 Nondestructive Testing Department, National Institute of R&D for Technical Physics, 47 D. Mangeron Blvd, 700050 Iasi, Romania; E-Mail: steigmann@phys-iasi.ro; 2 Faculty of Physics, Alexandru Ioan Cuza University, 11 Carol I Blvd, 700506 Iasi, Romania; 3 CRISMAT Laboratory, National Graduate School of Engineering, University of Caen on Normandy, 6 Marechal Juin Blvd, Caen 14050, France; E-Mail: alina.bruma@utsa.edu; 4 Department of Physics and Astronomy, University of Texas at San Antonio, One UTSA Circle, San Antonio, TX 78249, USA; 5 Faculty of Mechanical Engineering, University of Ljubljana, Aškerčeva 6, 1000 Ljubljana, Slovenia; E-Mail: roman.sturm@fs.uni-lj.si

**Keywords:** electromagnetic sensor, damages, metamaterials lens, FRPC, nondestructive evaluation

## Abstract

This paper proposes the study and implementation of a sensor with a metamaterial (MM) lens in electromagnetic nondestructive evaluation (eNDE). Thus, the use of a new type of MM, named Conical Swiss Rolls (CSR) has been proposed. These structures can serve as electromagnetic flux concentrators in the radiofrequency range. As a direct application, plates of composite materials with carbon fibers woven as reinforcement and polyphenylene sulphide as matrix with delaminations due to low energy impacts were examined. The evaluation method is based on the appearance of evanescent modes in the space between carbon fibers when the sample is excited with a transversal magnetic along z axis (TMz) polarized electromagnetic field. The MM lens allows the transmission and intensification of evanescent waves. The characteristics of carbon fibers woven structure became visible and delaminations are clearly emphasized. The flaws can be localized with spatial resolution better than λ/2000.

## 1. Introduction

In the past several decades, a number of nondestructive evaluation (NDE) techniques have been developed for detecting effect of damages/embedded objects in inhomogeneous media. Carbon fiber reinforced plastics (CFRP) are widely used in aeronautical industry [1,2] in flaps, ailerons, landing-gear doors, *etc*. Additionally, fiber reinforced polymer composites (FRPC) can be used in other areas like home construction, and naval, automotive and sport industry applications [3].

FRPC are classified, in general, as advanced and present continuous fiber reinforcement (for example, carbon, glass or aramide) of high modulus or high strength embedded in a thermoset or thermoplastic polymeric matrix. The appropriate performance of these composites during use is mainly related to their mechanical properties and thermal resistance as a result of the adequate combination of reinforcement (tapes or fabrics), polymeric matrix and processing techniques [3]. Polymeric composites reinforced with carbon reinforcements show mechanical properties similar or higher than those of conventional metallic materials, because an advanced polymeric composite presents high strength-to-weight and stiffness-to-weight ratios [4]. In addition, FRPC presents higher fatigue strength and higher corrosion resistance. Among the polymeric matrices, polyphenylene sulfide (PPS) is a versatile material that gives extruded and molded components the ability to meet exceptionally demanding criteria. Synthetic fibers and textiles derived from this polymer are known to resist chemical and thermal attack and the gas released due to ignition of matrix is substantially low [5], the effect of water absorption is reduced.

FRPC is being used in aerospace applications, because they have the required mechanical strength, chemical resistance and service temperature [6]. In order to be efficiently used, the polymer matrix of these composites must maintain its properties. Due to this reason, the FRPCs’ producers recommend a specific temperature interval in which the composite can function as designed. During the use of these composite structures, the maximum temperature can locally exceed certain values. This situation can take place in the case of structures used in aeronautics. Here, involving NDE methods is crucial to detect and emphasize these regions. The shape of defects in composites is very often different from those typically formed in metallic materials and therefore fracture mechanisms are much more complex due to the heterogeneous nature of composites [7]. Damage detection is a primary concern in composite structures, because damage can be hidden within the structure. Damage can include matrix cracking, fiber breakage and delamination, which can be caused by impacts, fatigue or overloading. The specific delaminations of FPRC due to impacts, even at low energies, or overloading of the structure can be detected by ultrasound procedures such as acoustic microscopy [8] and by different electromagnetic methods [9,10,11] these techniques allow the detection of flaws at depths up to six layers, including the possibility to obtain better resolution at test frequency [12]. When the composite gets elastically deformed local alteration of the electric conductivity occurs. At the same time, debonding on small zones of reinforcing fibers from the resin matrix can appear. For high-energy impacts, the local deformation results in delamination, deviation and/or breaking of carbon fibers. In this case, local modification of the electric conductivity occurs, which can be detected by electromagnetic nondestructive evaluation (eNDE) [13,14,15,16] and the debonding of carbon fibers from the resin matrix can be detected by these methods [17]. The delamination can also be clearly detected by means of other nondestructive methods, such as shearography and active thermography [18,19]. Visible damages can be clearly detected and repairing can be made in order to maintain structural integrity. But, a major problem consists is the growth of undetected, hidden damages caused by low velocity impacts. These damages are known in aerospace applications as Barely Visible Impact Damages (BVID) [20].

Irrespective of the evaluation methods utilized, the examination procedures should be effective, secure and should introduce no ambiguities and should be conducted at the highest possible control speed. Modern technologies of fabrication involve the use of new materials such as composites at large scale. This imposes developing of NDE techniques in order to reach performances required by the design.

The NDE of materials consists of the application of a physical field to the examined object and evaluating the interaction between the field and the eventual material discontinuities. If the physical field applied to the examined object is an electromagnetic field with frequencies ranging in interval tens of Hz to tens of GHz, the procedure is eNDE. This is applied to the examined having high conductivity, in which, under the action of electromagnetic incident field, eddy currents are induced, according to Faraday’s law [21]. The induced currents create a secondary electromagnetic field opposing the incident one. The presence of material inhomogeneities (voids, inclusions, cracks, with lower electrical conductivity) will disturb induced eddy currents and will change the apparent impedance of sensors.

At this time, eNDE of FRPC is carried out by using eddy current sensors with ferrite core probes [22,23] or pancake coils [24], with and without shielding and metamaterial (MM) sensors used for improvement of electromagnetic image quality of microscopic discontinuities in conductive pieces as FRPC composites as well as metallic strip gratings from flexible printed circuits can be made using evanescent modes generated in slits, in spaces between carbon fibers and respectively in cracks [25].

For relatively small frequencies, (<1 GHz), the incident electromagnetic field is created by a coil crossed by an alternative electric current [26] or pulsed currents [27], the detection of electromagnetic fields created by the secondary source being achieved by using coils of different shapes [28]. For low frequencies, the detection of the secondary electromagnetic field can be obtained with sensors with Hall effect and GMR sensors or SQUID [29].

In all cases it is understood that the signal to noise ratio is larger at detection for this is necessary to maintain the smallest lift-off (=distance between the sensor and surface to be examined) possible. Lord Rayleigh advanced an approach to diffraction calculation in his solution to wave scattering from a reflecting grating [30] when the size or periodicity of diffracting object becomes comparable to or smaller than the wavelength of the incident electromagnetic wave. This is obtained working in the near field [31] due to the fact that the generated and scattered electromagnetic waves are evanescent (=waves that are rapidly attenuated with distance [32]) and are difficult to be focalized using classical materials.

Loss-free metamaterials cannot be achieved, but with finite loss, imaging with sub-wavelength resolution can be achieved [33]. Working in the very near field, the electric and magnetic fields are essentially decoupled, so that a “poor-man’s perfect lens” can be made, in which a material with ε*_eff_* = −1 acts on the electric field, or with μ*_eff_* = −1 acts on the magnetic field. These two cases have been demonstrated using silver in the visible spectrum to achieve electric field imaging with λ/6 resolution [34,35] and using a classical Swiss roll medium in radiofrequency regime to image magnetic objects with resolution of λ/64 [36], the maximum resolution being limited by the metamaterial’s losses [33].

This paper presents a possibility to enhance the spatial resolution of eNDE methods that operate at frequencies of tens to hundreds of MHz using a sensor with a metamaterial lens constructed from two conical Swiss rolls. The possibility to manipulate the evanescent waves that appear in the space between carbon fibers allow for an improvement of the spatial resolution to at least λ/2000, exceeding the limit imposed by diffraction.

## 2. Electromagnetic Sensor with Metamaterial Lens

### 2.1. Theoretical Principles

#### 2.1.1. Constitutive Parameters of Conical Swiss Rolls

The electromagnetic sensor used in the eddy current microscopy must be an absolute send-receiver-type [37]. The field generated by the emission part of the sensor must be as focused as possible for frequencies in the range of tens of MHz, where the ferrites have a diminished relative permeability and great losses. The scattered field must be also focused such that it can be detected by the reception part of the sensor. Recently, NDE technologies based on MM sensors have attracted significant attention from the UHF to optical frequencies because of their cost-efficiency and better performance than traditional structures, due to their unique structural properties (high Q factors compared to the spiral structures, they can be embedded in the tested material and can assure structural monitoring at microscopic level [38,39]).

The MM [40,41] can provide an engineered response to the electromagnetic radiation that is not available in naturally occurring materials. These are often defined as the structures of metallic and/or dielectric elements, with periodic arrangements in three or two dimensions [41]. The size of the structures is typically smaller than the free space wavelength of the incoming electromagnetic waves. Nowadays, a multitude of MM structural elements type are known, conferring special electromagnetic properties [42,43]. In function of the incident electromagnetic field frequency, the type and geometrical shape, MM may have a high relative magnetic permeability either positive or negative [40]. These properties strongly depend on the geometry of MM rather than their composition [42] and experimentally demonstrated [43]. All researches reported until now, capitalize, in different manners the possibility to realize perfect lens. The MM lenses assure the possibility to apply of electromagnetic MM in eNDE. For a MM slab characterized by effective permittivity ε*_eff_* and effective magnetic permeability µ*_eff_*, the refractive index is:

(1) $$n=\sqrt{\varepsilon_{eff}\mu_{eff}}$$

and the impedance is given by:

(2) $$Z=\sqrt{\frac{\mu_{eff}}{\varepsilon_{eff}}}$$

The relation between S parameters and effective refractive index n is given by [44,45]:

(3a) $$S_{11}=\frac{R_{0_{1}}\left(1-e^{j2nk_{0}d}\right)}{1-R_{0_{1}}^{2}e^{j2nk_{0}d}}$$

(3b) $$S_{21}=\frac{\left(1-R_{0_{1}}^{2}\right)e^{j2nk_{0}d}}{1-R_{0_{1}}^{2}e^{j2nk_{0}d}}$$

where $R_{0_{1}}=\frac{Z-1}{Z+1}$ and the impedance *Z* is obtained by inverting Equations (3a) and (3b), yielding:

(4a) $$Z=\pm \sqrt{\frac{{\left(1+S_{11}\right)}^{2}-S_{21}^{2}}{{\left(1-S_{11}\right)}^{2}-S_{21}^{2}}}$$

(4b) $$e^{jnk_{0}d}=X\pm j\sqrt{1-X^{2}}$$

where $X=\frac{1}{2S_{21}\left(1-S_{11}^{2}+S_{21}^{2}\right)}$.

The connection between ε and µ for a medium as well as the wave propagation through can be categorized into four classes [46]. For double negative medium (DMG) when ε*_eff_ =* −1 and µ*_eff_ =* −1, the refractive index of MM slab is *n =* −1 [47] and the surface impedance *Z* = 1, such that there is no mismatch and consequently no reflection on the interface slab-air. This metamaterial slab forms perfect lens [46,48], and is focusing the electromagnetic field and also the evanescent waves. MM lenses are lenses that, at working frequency, have either ε*_eff_ =* −1 and then electric evanescent modes can be manipulated, either µ*_eff_ =* −1 and then the lens can focus magnetic evanescent modes [40].

#### 2.1.2. Functioning Principle of a MM Lens Sensor

**Figure 1:**
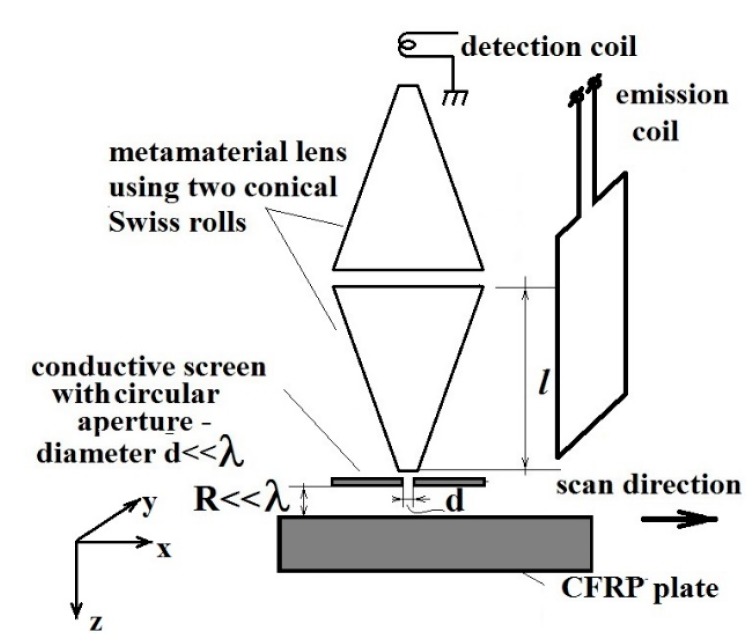
Schematic representation of the Sensor with MM lens.

Electromagnetic sensors with MM lenses have been made in our case using conical Swiss rolls (CSR) [49], the operation frequencies depending both by the constitutive parameters of MM as well as by the polarization of the incident electromagnetic field (TEz or TMz). Figure 1 shows a sensor with MM developed by us [50,51,52]. As shown in [49], the electrical evanescent modes can be manipulated with a lens realized with CSR, functioning in the range of frequencies such that µ*_eff_* is maximum. Moreover, working at frequency that assures µ*_eff_ =* −1 for the same lens, the magnetic evanescent modes can be focalized [31,32].

The principle scheme of the evanescent wave’s detection using a circular aperture with very small diameter and MM lens, CSR type is shown in Figure 1. The detection principle is similar with the one of near-field electromagnetic scanning microscopy (NFESM). NFSEM imaging is a sampling technique, *i.e.*, the sample (in our case FRPC) is probed point by point by raster scanning with the sensor over the sample surface and recording for energy image pixel a corresponding electromagnetic signature. The reception coil functions as a detection antenna, converting localized energy into an electromotive force. The focal distance of the lens using a MM is [49]:

(5) f≅l

where *l* is the height of a CSR. The functioning of the entire detection system can be described using Fourier optics [53,54].

Considering the FRPC as illuminated with a plane wave, TMz polarized, at normal incidence, the magnetic field being parallel with the *y* axis such that *H*_x_ = *H*_z_ = 0 and *H*_y_ ≠ 0, in the very near field, evanescent wave can appear between carbon fibers similar with metallic strip gratings [31,52] and the eigenmodes between the carbon fibers playing the role of object for the detection system from Figure 1. These waves can be manipulated using this type of electromagnetic sensor with MM lens.

The circular aperture with *d* diameter is introduced with the pupil function *P*(*x, y*) defined as:

(6) $$P\left(x,y\right)=\{\begin{array}{cc} 1 & x^{2}+y^{2}\leq d^{2}\\ 0 & otherwise \end{array}$$

At the output of the detection system, the image *I(x’, y’)* of the object *O(x, y)* is given by [46]:

(7) $$\begin{array}{l} I\left(x^{'},y^{'}\right)=\frac{1}{\lambda^{2}d_{1}d_{2}}\int_{-\infty }^{\infty }\int_{-\infty }^{\infty }\exp\left[i\frac{k\left({\left(x^{'}-x_{1}\right)}^{2}+{\left(y^{'}-y_{1}\right)}^{2}\right)}{2d_{2}}\right]P\left(x,y\right)\exp\left[i\frac{k\left(x_{1}^{2}+y_{1}^{2}\right)}{2f}\right]\times \\ \left(\int_{-\infty }^{\infty }\int_{-\infty }^{\infty }O\left(x,y\right)\exp\left[i\frac{k\left({\left(x_{1}-x\right)}^{2}+{\left(y_{1}-y\right)}^{2}\right)}{2d_{1}}\right]dxdy\right)dx_{1}dy_{1} \end{array}$$

where *f* is the focal distance of the lens equal with the height of CSR, λ is the wavelength in vacuum, k=2π/λ is the wave number, $d_{1}=R+l$ is the distance from the object to the center of the lens, $d_{2}=l$ is the distance from the center of the lens to the detecting coil.

**Figure 2:**
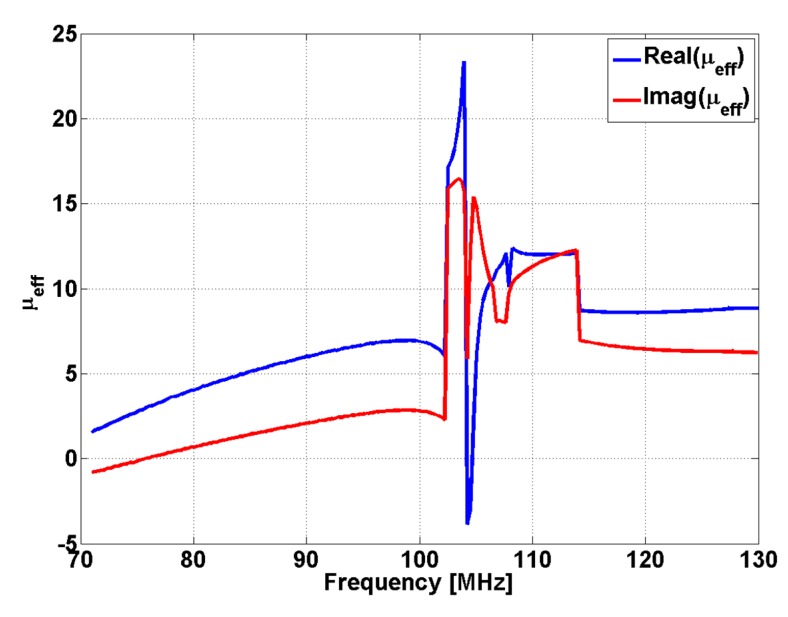
The frequency dependence of effective permeability of conical Swiss roll.

If we consider an electromagnetic sensor with MM lens having *f = l =* 55 mm, in front of which a conductive screen having a circular aperture with diameter *d =* 100 µm is placed, the effective permeability of the lens presents a maximum at 105 MHz (Figure 2). The frequency range for which the effective permeability is negative is extremely narrow, as observed in Figure 2. The effective permeability and permittivity can be determined by measuring reflection and transmission coefficients for normal incidence of the electromagnetic wave to material slab [55]:

(8) $$\begin{array}{l} S_{11}=R\\ S_{21}=T\exp\left(jKd\right) \end{array}$$

when R and T are reflection and respective transmission coefficient.

If $k^{2}>k_{x}^{2}+k_{y}^{2}$, the electromagnetic wave is a propagating plane wave, and if $k^{2}<k_{x}^{2}+k_{y}^{2}$, the electromagnetic wave is an evanescent wave, being rapidly damped along z axis. Consider the propagation nearly parallel to the z axis, (paraxial approximation), situation in which the operation mode of the metamaterial lens can be explained using Fourier optics principles [53].

According to [31], the field in focal plane is:

(9) $$H\left(x,y,z_{0}+2l\right)=\frac{H_{0}}{\pi^{2}}e^{j\left(\frac{k_{b}+k_{a}}{2}x+\frac{k_{b}+k_{a}}{2}y\right)}\frac{\sin\left(\frac{k_{b}-k_{a}}{2}x\right)}{x}\frac{\sin\left(\frac{k_{b}-k_{a}}{2}y\right)}{y}$$

where *z*_0_ = R (Figure 1a) with R << λ, *H*_0_ is amplitude of incident magnetic field.

Circular aperture made from perfect electric conductor PEC material is used in order to obtain relatively uniform angular spectrum due to the scattering on this aperture, assuring paraxial incident beam.

The diameter of focal spot provided by MM lens is given by [53]:

(10) $$D=\frac{4\pi }{k_{b}-k_{a}}$$

and is equal with the diameter of the small basis of the conical Swiss roll, *i.e.*, 3.2 mm. The MM lens with CSR will be displaced along the *x*-axis (Figure 1). From this reason *k_a_* = 0 and inserting this value in Equation (10), *k_b_* is obtained and the field in focal plane is calculated as in Equation (9).

### 2.2. Physical Realization of MM Lens Sensor

**Figure 3:**
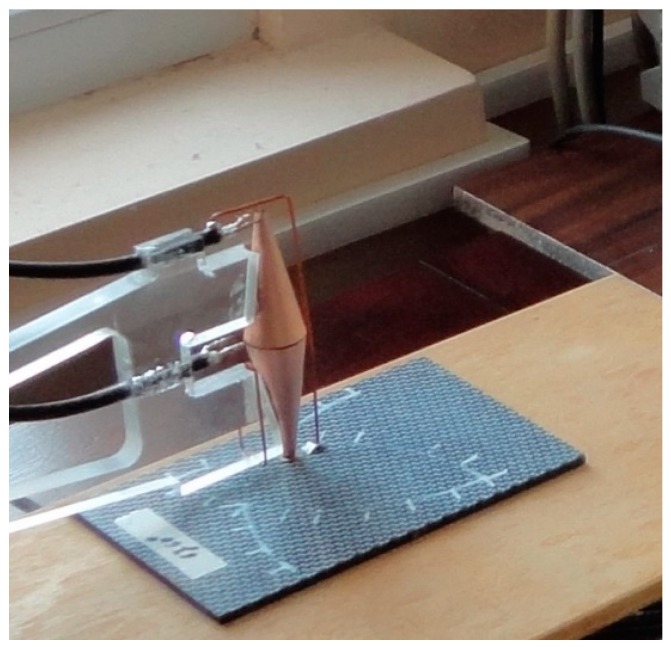
Design of device for testing.

The MM lens has been realized with two CSRs having a large basis face to face (Figure 1). A CSR consists of a number of spiral wound layers of an insulated conductor on a conical mandrel. CSRs are tuned at 105 MHz frequency. The geometrical parameters of CSR are 20 mm base diameter, 3.2 mm top diameter, the aperture angle 20°, height of 55 mm and 3 turns made from 18 µm thickness copper foil adhesiveless laminated with 12 µm thickness polyimide foil (LONGLITE™200 produced by Rogers Corporation (Connecticut, CT, USA), in order to decrease the losses at high frequencies (Figure 3). The Swiss rolls act like MM allowing the transmission of the evanescent waves from the region between carbon fibers towards the reception coil placed in the focal image point**,** converting the localized energy in electromotive force (e.m.f.).

The incident field is generated by a one-turn rectangular coil, having 35 × 70 mm, using a Cu wire with 1 mm diameter. The reception coil has one turn with 3 mm average diameter made of Cu wire with 1 mm diameter. In Figure 2 was present the dependency by frequency of the effective magnetic permeability of the lens used for manipulation of evanescent waves. It can be observed that the effective magnetic permeability of the lens became high for a certain frequency range and it becomes negative for other frequency range. At a resonance frequency of 105 MHz, the relative magnetic permeability is 24.

**Figure 4:**
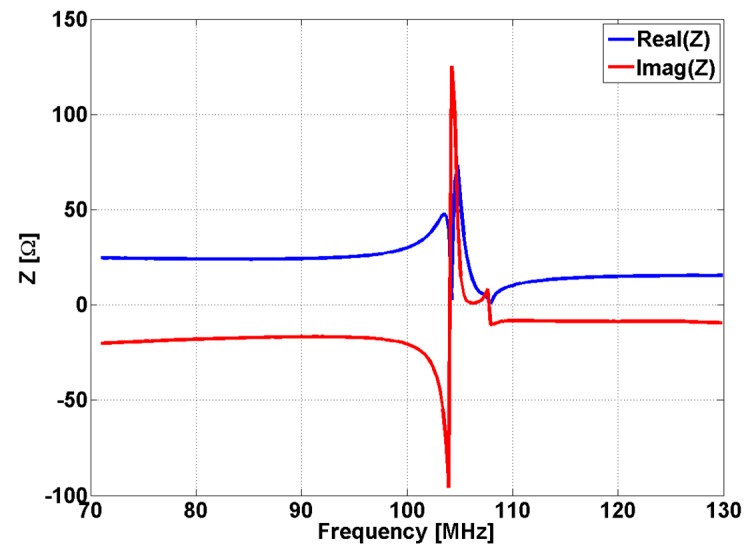
The dependency by frequency of CSR impedance.

The dependency of frequency for the effective magnetic permeability of a CSR has been determined measuring the S parameters (S_11_ and S_21_) and applying the effective medium method [56,57] using a 4395A Network/Spectrum/Impedance Analyzer Agilent (Agilent Technologies, Santa Clara, CA, USA) coupled with an Agilent 87511A S Parameters Test kit . The dependence by frequency of impedance of CSR given by S parameters [57] in Equation (4a) is presented in Figure 4.

**Figure 5:**
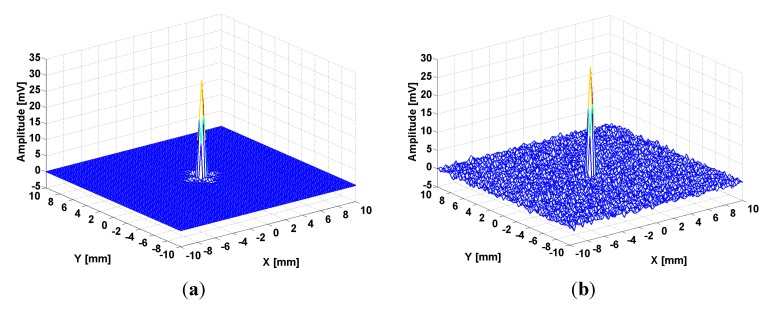
Point spread function for MM lens: (**a**) calculated; (**b**) measured.

The spatial resolution of the system (= the distance between two distinctively visible points) was verified on a test block made from Plexiglas of 8 mm thickness [49], containing in its center a 1 mm diameter borehole in which a copper cylinder was inserted, by simulation (Figure 5a) as well as by experimental measurement of the value of e.m.f. from scattering on the base of the Cu cylinder (Figure 5b). The Cu cylinder can be considered as a point scatterer so that the response of the electromagnetic system at the scanning of the region which contains the scatterer represents the point spreading function for the sensor with the MM lens [53].

The Plexiglas plate with the Cu cylinder are displaced with a XY displacement system Newmark Systems Inc. (Santa Margarita, CA, USA), in front of the sensor with the MM lens, maintained in a fixed position. The scanning step was 0.25 mm in both directions. In order to maximize the value of effective magnetic permeability of MM lens, the working frequency used in measurements with the Agilent 4395A Network/Spectrum/Impedance Analyzer was 105 MHz. Super resolution is obtained due to the manipulation of evanescent waves using the MM lens, reaching a value of approximately λ/2000.

## 3. Studied Samples and Experimental Set-Up

The study involved quasi-isotropic FRPC plates made by Tencate (Almelo, The Netherlands) [5], having 150 × 100 × 4.2 mm^3^, containing 12 layers of five harness satin carbon fibers woven with balanced woven fabric [58]. The matrix is made of polyphenylene sulphide (PPS), a thermoplastic polymer consisting of aromatic rings linked with sulphide moieties, resistant to chemical and thermal attack, and the amount of gas released due to matrix ignition is substantially low. The carbon fibers are T300JB type and their volume is 0.5 ± 0.03 and the density is 1460 kg/m^3^.

**Figure 6:**
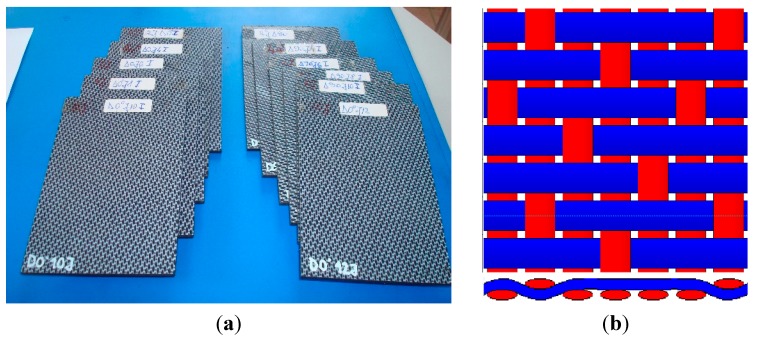
Studied samples: (**a**) Photo; (**b**) 5HS woven layout.

Figure 6 presents the studied samples and the layout of five harness satin woven of carbon fibers. The plates were subjected to impacts with energies of 2, 4, 6, 8, 10 and 12 J. The composite plates exhibit electric properties that depend on the type of carbon fibers and on their volume fraction in the material, having the transverse electric conductivity between 10 S/m and 100 S/m and longitudinal conductivity ranging between 5 × 10^3^ S/m and 5 × 10^4^ S/m. The samples were impacted using a FRACTOVIS PLUS 9350-CEAST instrument (Instron, Norwood, MA, USA) with a hemispherical bumper head having 20 mm diameter and 2.045 kg weight, in order to induce delaminations. The impact data were recorded with a DAS16000 acquisition system (Instron) with a sampling frequency of 1 MHz [59]. For higher energy impacts, local deformations result in delamination propagation, deviation and/or breaking of the carbon fibers. In both cases, the modification of local conductivity allows the damage detection using electromagnetic methods [60]. Typical records of force *vs*. time during impact can give information about the CFRP status (delaminated or not) [61]. Thus, only plates that show delamination were used in the study, meaning the plates impacted with 6 J, 8 J, 10 J and 12 J.

**Figure 7:**
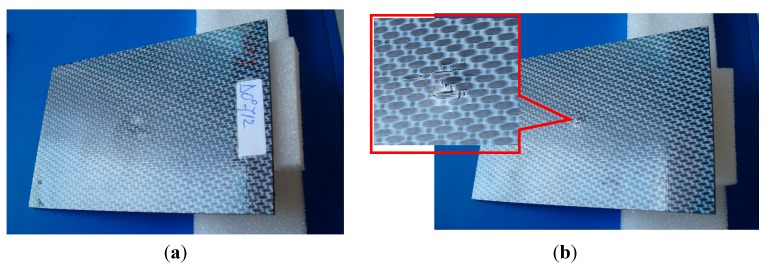
Sample impacted with 12 J energy: (**a**) front side; (**b**) back side—inset: breaking of fibers.

In Figure 7, images of both the plate faces impacted with 12 J energy are presented. Monitoring the strain in the transverse direction of FRPC in a laminate configuration is indeed an essential issue. In such structures, reinforcement fibers enhance the mechanical strength of composite in the plane of the structure but they suffer from fragility in the transverse direction.

The carbon fibers of FRPC need to be “illuminated” with a TMz wave to obtain the evanescent waves. The control of the distance between the surface of the plate and the conductive screen with circular aperture is important for an improvement of the spatial resolution. According to [50], the manipulation of evanescent waves can be performed with a MM lens. A conductive screen with a circular aperture having a diameter *d* = 100 µm was placed near the focal point at a distance of 75 µm from the surface to be inspected, which modifies the transmission through the lens and improves its quality without reducing the value of the S_21_ parameter considerably.

The composite plates were fixed on a Newmark X-Y displacement system that assures the displacement in plan with ±10 µm precision and rotation with ±2″. That assures the scanning of 60 × 60 mm^2^ with 1 mm steps in both directions. Electromagnetic inspection was made with sensors with MM lenses, the frequency control is 105 MHz and the distance from the bottom of the sensor, represented by the circular aperture to the surface to be inspected, is 75 µm. Once the level of noise was estimated, the optimal signal processing method is started. The scanning, the measurements, the acquisition and the processing of data were commanded by a PC using programs developed in Matlab 2011b. The performances of the sensor with MM were verified using the same mock-up used in [49].

The signal are generated and processed by the 4395A Agilent Impedance/Spectrum/Analyzer coupled through IEEE 488 interfaces with a PC which controls the displacement system. To obtain a superior resolution, in front of the sensor with MM lens a circular aperture has been used (according to Section 2 presented above).

## 4. Experimental Results

**Figure 8:**
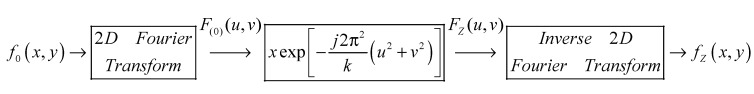
The image through an aperture due Fresnel diffraction.

At the scanning of the selected region of sample, the image delivered by the assembly sensor-equipment is amplified. This is due to the diffraction on the aperture of the evanescent waves generated by scattering on the woven carbon fibers. This assure that the structure of woven carbon fibers became clearly visible. Considering an object placed in the plane *z* = 0 and described by the function *f*_0_(*x*, *y*), at passing through an aperture, in the case of Fresnel diffraction (*i.e.*, when the aperture is close to the object), the image obtained at the distance *z* from the object will be *f*_z_(*x*, *y*) and can be calculated using the algorithm presented in Figure 8, according to the principle of Fourier optics [54]:

To ensure that *f_z_*(*x*, *y*) might represent exactly the figure of Fresnel diffraction through the radius aperture, between the Fourier variables and the spatial ones there must exist the relation:

(11) $$du=\frac{2a}{Ndx_{0}}$$

(12) $$dv=\frac{2a}{Mdy_{0}}$$

where *N* and *M* represent the maximum number of measurement points along the *x* and *y* directions and *dx_0_* and *dy_0_* are the scanning steps along the *x* and *y* directions, respectively.

Inverting the operation from Figure 8, the object *f_0_*(*x*, *y*) can be determined knowing the diffraction figure *f_z_*(*x*, *y*)*.* The measurements effected with the aperture make the recorded signals represent *f_z_*(*x*, *y*)*.* Applying the procedure described above, the shape of the object that scatters the electromagnetic field created by the emission coil can be determined.

**Figure 9:**
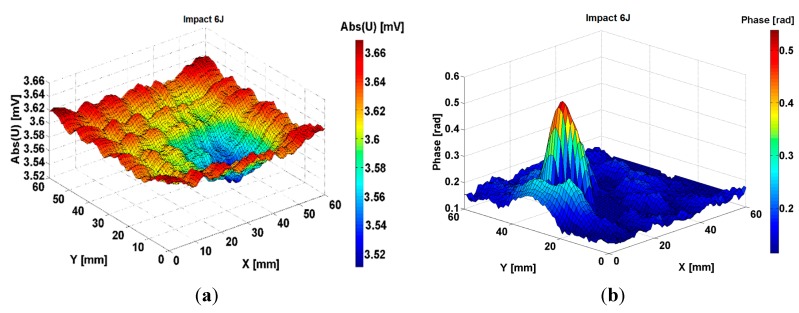
The measured signal delivered by the electromagnetic sensors with MM lens at scanning of composite FRPC quasi-isoptropic in plane samples impacted with 6 J energy (**a**) amplitude; (**b**) phase.

From the mechanics of composite materials it is known that due to an impact normal to the surface of the composite, delaminations can appear, whose shapes are approximately concave, a fact visible in Figure 9a. Therefore, the area of the delaminated surface can be determined, making the method effective for the examination of FRPC. In Figure 9a,b we show the information regarding the amplitude and phase for the signal induced in the reception coil of the sensor by the scanning of a region 60 × 60 mm^2^ of the composite, which contains a delamination following an impact of 6 J energy. On the border of the electromagnetic image, the structure of the woven fabric can be observed, while in the central zone, the delaminated region is emphasized.

Analyzing the phase information for composites impacted with the energies between 6 J and 12 J, these are completely changed compared to the non-impacted composite. The layout of the carbon fibers cannot be observed from the phase information but an important modification of the phase of e.m.f. induced in the reception coil can be seen and is presented below.

**Figure 10:**
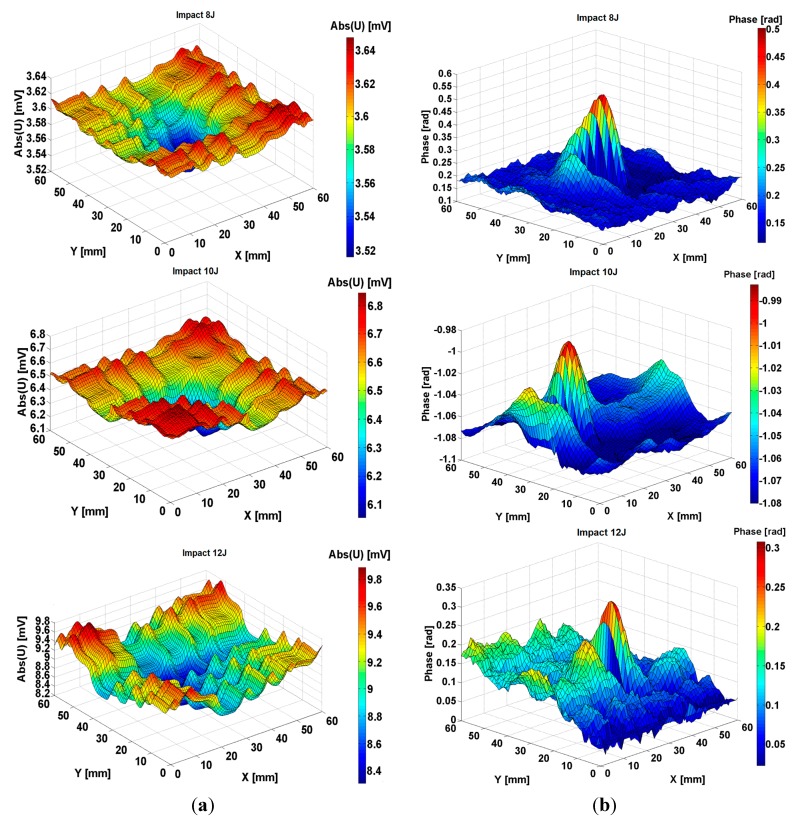
The measured signal delivered by the electromagnetic sensors with MM lens at scanning of composite FRPC quasi-isoptropic in plane samples impacted with 8 J, 10 J and 12 J energy (**a**) amplitude; (**b**) phase.

The method presents the advantage of giving in one representation both the information about amplitude and phase obtained from the sensor. This zone becomes electromagnetically detectable due to the modification of the electrical conductivity on the transversal direction to the woven fabric plane as consequence of the impact.

Figure 10 presents the signal given by the same sensor at the scanning of a region of composite which contains a delamination due to 8 J, 10 J and 12 J energy impact. Examining Figure 9 and Figure 10, it can be observed that for impacts with energies equal to 6 J and higher, the energy absorbed by the composite increases with the increasing impact energy, so the area of delamination increases in the same manner. It is visible that plastic deformations appear in the region of impact that lead to an increasing electrical conductivity of the area. Fibers in electrical contact, debonding of fibers from matrix can appear as consequences of the mechanical strength of the structures made from this composite.

eNDE using sensors with MM lenses is an emerging ND technique which combines the advantages of conventional eddy current testing and evanescent waves detection giving a higher resolution than eddy current. Our results aim to identify the location and approximate the dimensions of the damage in future work.

## 5. Conclusions

In order to detect the intensification of evanescent waves, a MM lens was developed using CSR for which an optimal working frequency was chosen so that the magnetic permeability shall be maximum and it also concentrates the magnetic flux in the radiofrequency domain, without being disturbed by a continuous intense magnetic field. The constitutive parameters of MM are determined using S parameters. A special attention is granted to MM lenses based on conical Swiss rolls configurations, for an optimal frequency which assures the concentration of the incident electromagnetic field and the evanescent waves can be effectively manipulated.

The performances of the electromagnetic sensors with MM lens can be improved, regarding the sensitivity and the spatial resolution, using the evanescent wave that can appear in the space between carbon fibers as FRPC composite structures excited with a plane TMz polarized electromagnetic wave.

This type of sensor allows one to obtain information about the amplitude and phase of signals induced in the reception coil of sensor while scanning a region of composite which contains a delamination and about local modifications of the transverse electric conductivity in FRPC materials, as the results of impact. In this way, the possibility to evaluate the state of the woven fabric and the damages due to impacts with different energies can be substantially improved.

The delamination with low energies is netly visible from the signal due to the woven fabric structure, the energy absorbed by the composite increases with the increase of the impact energy, so the area of the delamination increases in the same manner. The use of evanescent waves and lenses with metamaterials allows increasing the spatial resolution, due to the possibility of manipulating the evanescent waves, by approximately λ/2000.

## References

[1] Schwartz M.M. (1997). Composite Materials: Properties, Non-Destructive Testing, and Repair.

[2] Morgan P. (2005). Carbon Fibers and Their Composites.

[3] Pilato L.A., Michno M.J. (1994). Advanced Composite.

[4] Landel R.F., Nielsen L.E. (1993). Mechanical Properties of Polymers and Composites.

[5] TenCate Advanced Composites. http://www.tencate.com/.

[6] Greisel M., Jäger J., Moosburger-Will J., Sause M.G.R., Mueller W.M., Horn S. (2014). Influence of residual thermal stress in carbon fiber-reinforced thermoplastic composites on interfacial fracture toughness evaluated by cyclic single-fiber push-out tests. Compos. Part A.

[7] Elmarakbi A., Mollón V., Bonhomme J., Viña J., Argüelles A. (2013). Advanced Composite Materials for Automotive Applications: Structural Integrity and Crashworthiness.

[8] Gros X.E., Ogi K., Takahashi K. (1998). Eddy Current, Ultrasonic C-Scan and Scanning Acoustic Microscopy Testing of Delaminated Quasi-Isotropic CFRP Materials: A Case Study. J. Reinf. Plast. Compos..

[9] Lemistre M., Gallaud C., Gouyon R., Balageas D. Une methode magnetique radiofrequence de localisation des defauts dans les structures en composite carbone. Proceedings of the Congress Cofrend 97 sur les Essais non Destructifs.

[10] Grimberg R., Savin A., Radu E., Mihalache O. (2000). Nondestructive evaluation of the severity of discontinuities in flat conductive materials by an eddy-current transducer with orthogonal coils. IEEE Trans. Magn..

[11] Mook G., Michel F., Simonin J. (2011). Electromagnetic imaging using probe arrays. Strojniški Vestnik J. Mech. Eng..

[12] Schulze M.H., Meyendorf N., Heuer H. (2010). Analysis Techniques for Eddy Current Imaging of Carbon Fiber Materials. Mater. Test..

[13] Grimberg R., Premel D., Savin A., le Bihan Y., Placko D. (2001). Eddy current holography evaluation of delamination in carbon-epoxy composites. INSIGHT.

[14] Gros X.E. (1996). Some Aspects of Electromagnetic Testing of Composites. INSIGHT.

[15] Heuer H., Schulze M., Pooch M., Gäbler S., Nocke A., Bardl G., Petrenz S. (2015). Review on quality assurance along the CFRP value chain—Non-destructive testing of fabrics, preforms and CFRP by HF radio wave techniques. Compos. Part B Eng..

[16] Mizukami K., Mizutani Y., Todoroki A., Suzuki Y. Detection of delamination in thermoplastic CFRP weld parts using eddy current testing and induction heating. Proceedings of the 11th European Conference on Non-Destructive Testing (ECNDT 2014).

[17] Davis C.W., Nath S., Fulton J.P., Namkung M. (1995). Combined investigation of eddy current and ultrasonic techniques for composite materials NDE. Rev. Prog. Quant. Nondestruct. Eval..

[18] Hung Y.Y., Chen Y.S., Ng S.P., Liu L., Huang Y.H., Luk B.L., Ip R.W.L., Wu C.M.L., Chung P.S. (2009). Review and comparison of shearography and active thermography for nondestructive evaluation. Mater. Sci. Eng. R.

[19] Usamentiaga R., Venegas P., Guerediaga J., Vega L., Molleda J., Bulnes F.G. (2014). Infrared thermography for temperature measurement and non-destructive testing. Sensors.

[20] Staszewski W.J., Mahzan S., Traynor R. (2009). Health monitoring of aerospace composite structures—Active and passive approach. Compos. Sci. Technol..

[21] Bladel J. (2007). Electromagnetic Fields.

[22] Cacciola M., Calcagno S., Megali G., Pellicano D., Versaci M., Morabito F.C. (2009). Eddy current modeling in composite materials. PIERS Online.

[23] Xu X., Liu M., Zhang Z., Jia Y. (2014). A Novel High Sensitivity Sensor for Remote Field Eddy Current Non-Destructive Testing Based on Orthogonal Magnetic Field. Sensors.

[24] Grimberg R., Savin A., Radu E., Chifan S. (2000). Eddy current sensor for holographic visualization of material discontinuities. Sens. Actuators A Phys..

[25] Savin A., Faktorová D., Pápežová M., Steigmann R. Electromagnetic nondestructive evaluation using metamaterials sensor. Proceedings of 10th International Conference ELEKTRO 2014.

[26] Miorelli R., Reboud C., Lesselier D., Theodoulidis T. (2012). Fast simulation method of multiple narrow cracks in planar stratified media. Electromagnetic Non-Destructive Evaluation (XV).

[27] Mandache C., Lefebvre J.H.V. (2006). Transient and harmonic eddy currents: Lift-Off point of intersection. NDT & E Int..

[28] Sophian A., Tian G.Y., Taylor D., Rudlin J. (2001). Electromagnetic and eddy current NDT: A review. INSIGHT.

[29] Bonavolontà C., Valentino M., Marrocco N., Pepe G.P. (2009). Eddy Current Technique Based on SQUID and GMR Sensors for Non-Destructive Evaluation of Fiber/Metal Laminates. IEEE Trans. Appl. Supercond..

[30] Sommerfeld A. (2004). Mathematical Theory of Diffraction.

[31] Grimberg R., Tian G.Y. (2012). High Frequency Electromagnetic Non-destructive Evaluation for High Spatial Resolution using Metamaterial. Proc. R. Soc. A.

[32] Grbic A., Eleftheriades G.V. (2003). Growing evanescent waves in negative-refractive-index transmission-line media. Appl. Phys. Lett..

[33] Grbic A., Eleftheriades G.V. (2004). Overcoming the Diffraction Limit with a Planar Left-Handed Transmission-Line Lens. Phys. Rev. Lett..

[34] Blaikie R.J., Melville D.O. (2005). Imaging through planar silver lenses in the optical near field. J. Opt. A Pure Appl. Opt..

[35] Fang N., Lee H., Sun C., Zhang X. (2005). Sub-Diffraction-Limited optical imaging with a silver superlens. Science.

[36] Wiltshire M.C.K., Pendry J.B., Hajnal J.V. (2006). Sub-Wavelength imaging at radio frequency. J. Phys. Condens. Matter.

[37] Grimberg R., Savin A., Rotundu C.R. (2001). Eddy current microscopy applied to graphite-epoxy composite. Sens. Actuators A Phys..

[38] Ozbey B., Unal E., Ertugrul H., Kurc O., Puttlitz C.M., Erturk V.B., Altintas A., Demir H.V. (2014). Wireless Displacement Sensing Enabled by Metamaterial Probes for Remote Structural Health Monitoring. Sensors.

[39] Savin A., Steigmann R., Dobrescu G.S. Metamaterial Sensors for Structural Health Monitoring. Proceedings of the ASME 2014 12th Biennial Conference on Engineering Systems Design and Analysis.

[40] Pendry J.B., Holden A.J., Robbins D.J., Stewart W.J. (1999). Magnetism from conductors and enhanced nonlinear phenomena. IEEE Trans. Microw. Theory Tech..

[41] Wiltshire M.C.K. (2007). Radio frequency (RF) metamaterials. Phys. Status Solidi B.

[42] Cai W., Chettiar U.K., Kildishev A.V., Shalaev V.M. (2007). Optical cloaking with metamaterials. Nat. Photonics.

[43] Smith D.R., Pendry J.B., Wiltshire M.C. (2004). Metamaterials and negative refractive index. Science.

[44] Shelby R.A., Smith D.R., Nemat-Nasser S.C., Schultz S. (2001). Microwave transmission through a two-dimensional, isotropic, left-handed metamaterial. Appl. Phys. Lett..

[45] Chen X., Grzegorczyk T.M., Wu B.I., Pacheco J., Kong J.A. (2004). Robust method to retrieve the constitutive effective parameters of metamaterials. Phys. Rev. E.

[46] Engheta N., Ziolkowski R.W. (2006). Electromagnetic Metamaterials: Physics and Engineering Explorations.

[47] Veselago V.G. (1968). The electrodynamics of substances with simultaneously negative values of ϵ and μ. Physics-Uspekhi.

[48] Pendry J.B. (2000). Negative Refraction Makes on Perfect Lens. Phys. Rev. Lett..

[49] Grimberg R., Savin A., Steigmann R., Serghiac B., Bruma A. (2011). Electromagnetic non-destructive evaluation using metamaterials. INSIGHT.

[50] Grimberg R., Savin A., Steigmann R. (2012). Electromagnetic imaging using evanescent waves. NDT&E Int..

[51] Grimberg R., Savin A. Electromagnetic Transducer for Evaluation of Structure and Integrity of the Composite Materials with Polymer Matrix Reinforced with Carbon Fibers. Romanian Patent.

[52] Savin A., Steigmann R., Bruma A. (2014). Metallic Strip Gratings in the Sub-subwavelength Regime. Sensors.

[53] Born M., Wolf E. (1975). Principle of Optics.

[54] Goodman J.W. (2005). Introduction to Fourier Optics.

[55] Smith D.R., Schultz S., Markoš P., Soukoulis C.M. (2002). Determination of effective permittivity and permeability of metamaterials from reflection and transmission coefficients. Phys. Rev. B.

[56] Kong J.A. (2000). Electromagnetic Wave Theory.

[57] Grimberg R. (2013). Electromagnetic metamaterials. Mater. Sci. Eng. B.

[58] Akkerman R. (2006). Laminate mechanics for balanced woven fabrics. Compos. Part B Eng..

[59] Steigmann R., Savin A., Katalinic B. (2014). Advanced Sensor for Enhancement of Electromagnetic Imaging of Impacted Carbon Fibers-PPS Composites.

[60] Menana H., Féliachi M. (2011). Electromagnetic characterization of the CFRPs anisotropic conductivity: Modeling and measurements. Eur. Phys. J. Appl. Phys..

[61] Ullah H., Abdel-Wahab A.A., Harland A.R., Silberschmidt V.V. (2012). Damage in woven CFRP laminates subjected to low velocity impacts. J. Phys.: Conf. Ser..

